# Supplementary material for: Integrated artificial neurons from metal halide perovskites
Source: Mater Horiz. 2025 Jan 20;12(8):2701–8. doi: 10.1039/d4mh01729c (PMC11745301; doi:10.1039/d4mh01729c)
Supplement: MH-012-D4MH01729C-s001 [file MH-012-D4MH01729C-s001.pdf]

Supplementary Information (SI) for Materials Horizons.
This journal is © The Royal Society of Chemistry 2024

Supplementary Information for

Integrated Artificial Neurons from Metal Halide Perovskites

Jeroen J. de Boer[1], Bruno Ehrler[1,*]

[1]Center for Nanophotonics, AMOLF, 1098 XG, Amsterdam, the Netherlands

[*]Correspondence: b.ehrler@amolf.nl

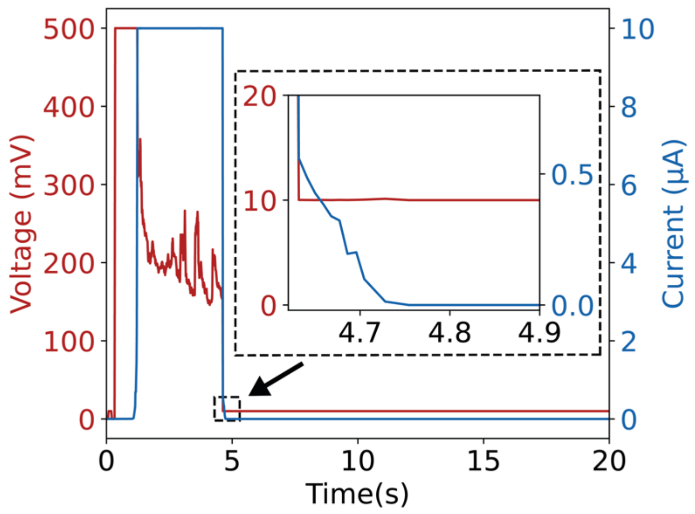

Figure S1. Retention time measurement of the resistive switch. A 500 mV pulse is applied to the device to bring the device to the low resistance state. After approximately one second, the resistance of the device rapidly drops. The measurement setup then reduces the applied voltage to ensure that the set compliance current of 10 μA is not exceeded. After 4.5 seconds, the voltage is reduced to 10 mV to measure the evolution of the resistance over time. The inset shows a zoom-in on the region in the dotted rectangle, corresponding to the first hundreds of milliseconds after the potential is reduced to the 10 mV read-out voltage.

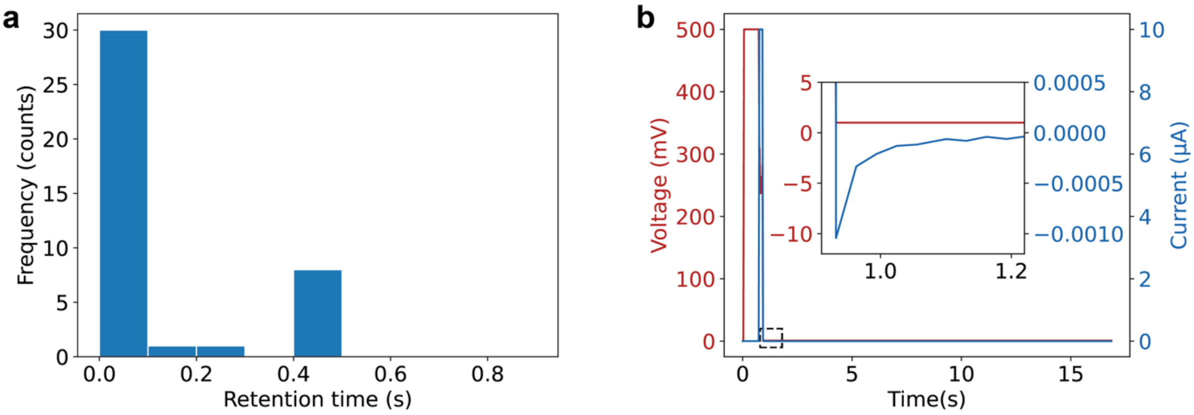

Figure S2. Analysis of retention times of the resistive switch. ***(a)*** *Histogram of 40 retention time measurements.* ***(b)*** *Example of a measurement where the retention time was less than the integration time of the measurement setup, so only a discharge of the parasitic capacitance is measured. Although the exact retention time of these measurements could not be determined, they were added to the first bin of the histogram to still give an accurate representation.*

Figure S2a gives a histogram of retention times based on 40 measurements. The device is set to the high-conductive state by applying 500 mV, with the compliance current set to 10 μA. The retention time was measured by applying 1 mV of constant bias. In several cases, the device reset to the low-conductance state within the integration time of the measurement setup (about 25 ms). An example of such a measurement is given in Figure S2b. These measurements were binned in the first bin (between 0 and 0.05 s) of the histogram in Figure S2a. The retention time was under 500 ms for all cases measured.

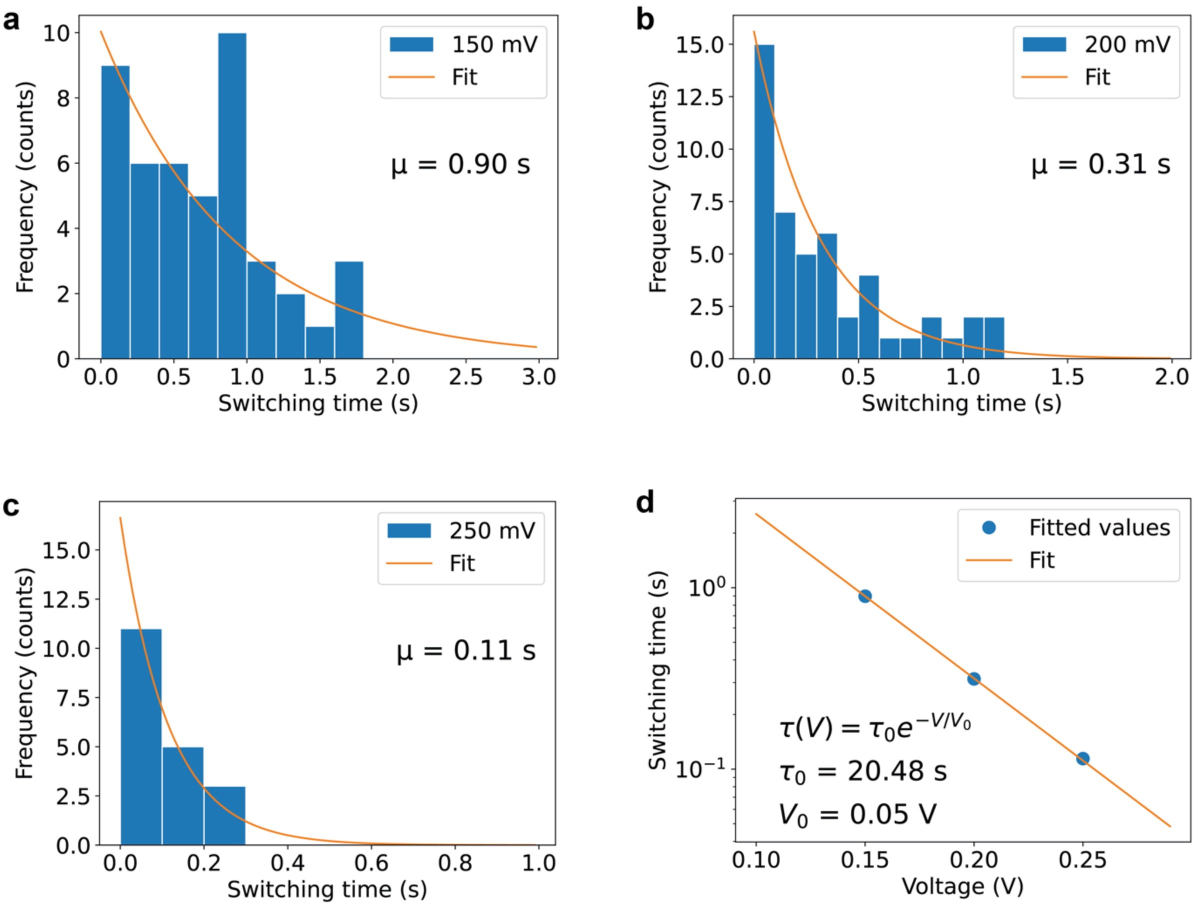

Figure S3. Histogram of the switching time for a switching event of the halide perovskite memristive device under an applied voltage of 150 ***(a)****, 200* ***(b)****, and 250 mV* ***(c)****, with a fit based on a Poisson distribution. The means obtained from the fits are given in their respective figures.* ***(d)*** *A fit of the means obtained in the previous subfigures to the listed exponential function to extract the fitting parameters.*

The probability of a resistance change of the memristive device upon the application of a voltage follows a Poisson distribution, as is evident from Figure S3a, b, and c. The formation of conductive filaments in memristive devices requires hopping of ions by a thermally activated process, which introduces this stochasticity. Random fluctuations are not averaged out according to the law of large numbers due to the small amount of ions needed to form the nanoscale filament. Previous work has described this Poisson behavior extensively and showed that the mean switching time depends exponentially on the applied bias according to $\tau(V) = \tau_0 e^{-V/V_0}$, where τ is the mean switching time, and τ_0 and V_0 are fitting parameters.[1] Figure S3d shows the same trend for our device, indicating that the same process of stochastic conductive filament formation underlies the operation of our device. Previous research on devices with similar electrodes and a

halide perovskite active layer has shown that these conductive filaments consist of iodide vacancies[2] or silver.[3]

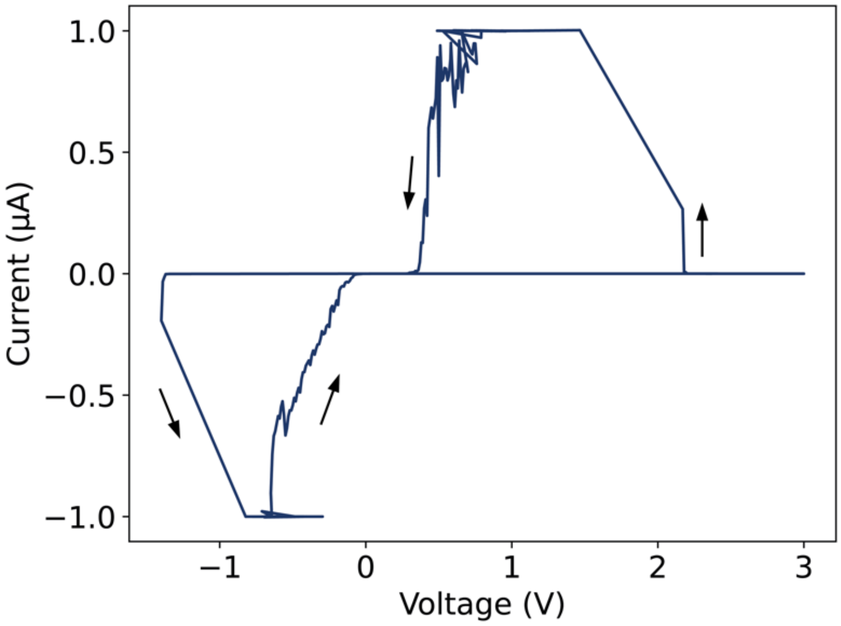

Figure S4. Resistive switching of a device without the halide perovskite layer. Resistive switching also occurs through the SiO_2 spacer, albeit at higher voltages than for the device with a halide perovskite layer.

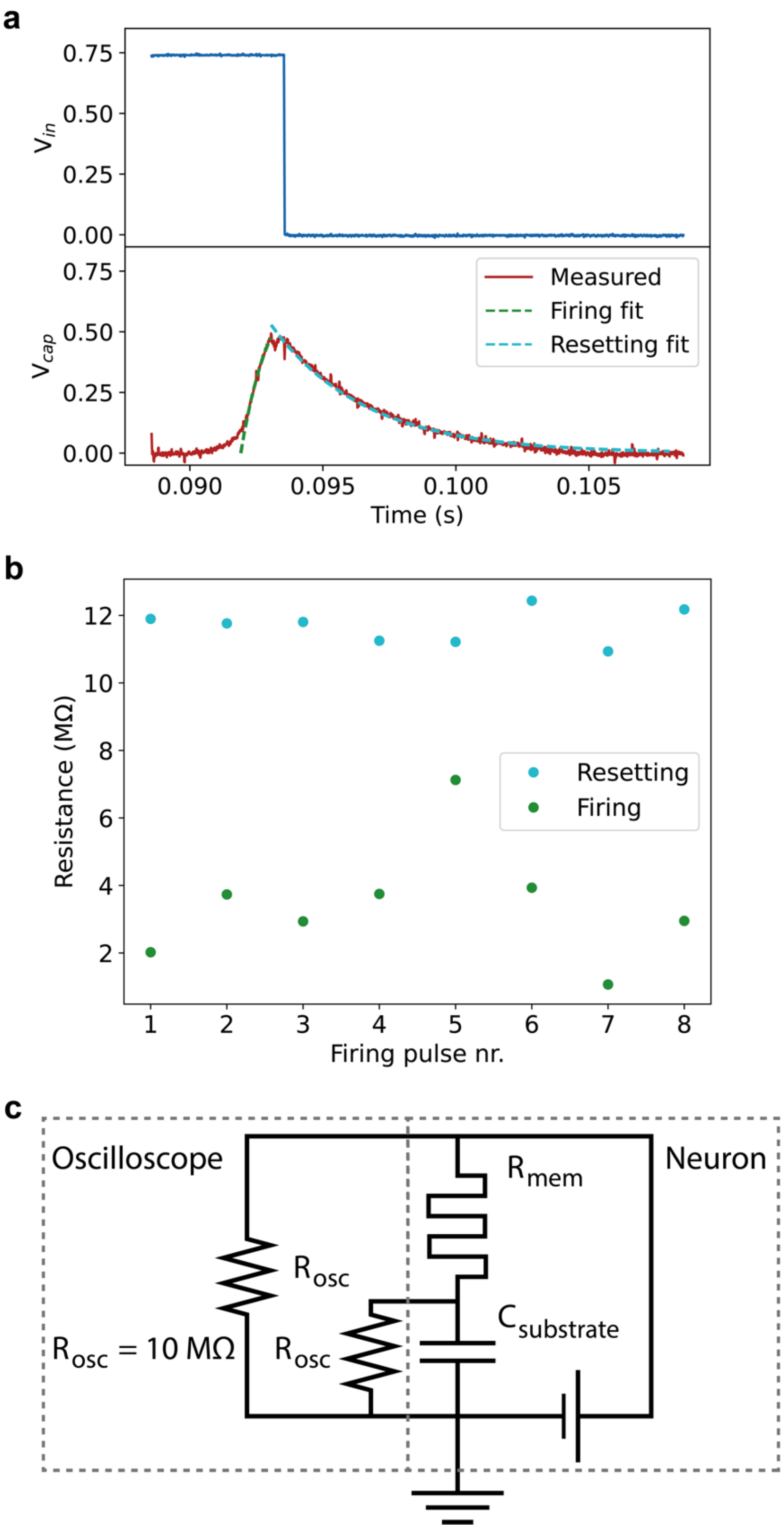

Figure S5. Fit of the firing pulses in Figure 2c with charging and discharging of a capacitor. ***(a)*** *Fits of the charging (firing) and discharging (resetting) of the second firing pulse.* ***(b)*** *Extracted resistances of the charging and discharging of each of the firing pulses in Figure 2c. Error bars representing one standard deviation of the obtained resistance from the fit are included, but are smaller than the dots of the markers in the figure.* ***(c)*** *The*

circuit of the neuron with the 10 MΩ probes of the oscilloscope connected. The probes offer an alternative path for the capacitor to discharge.

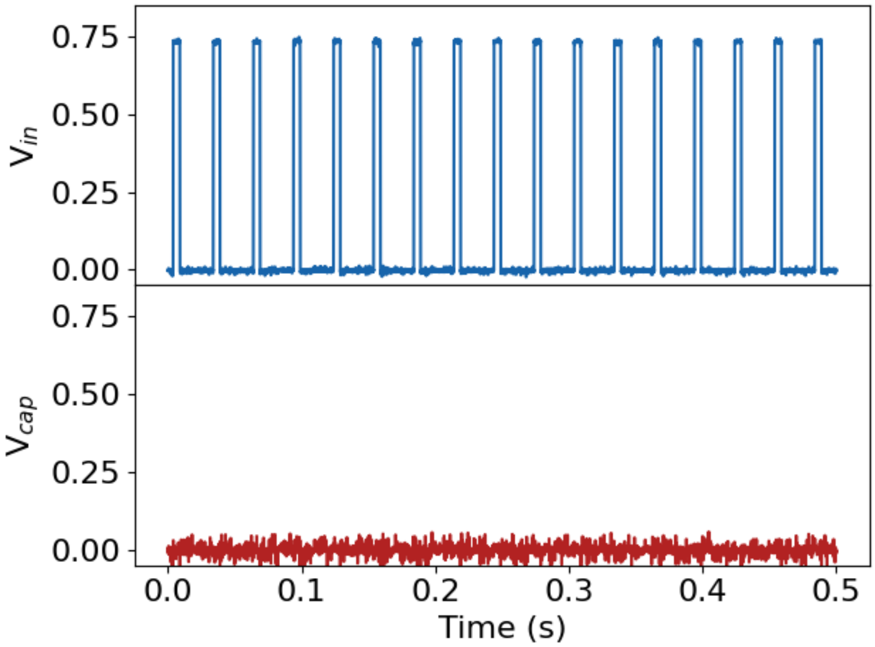

Figure S6. Spiking neuron measurements repeated on a substrate without the halide perovskite layer. No spiking is measured using the same parameters as for the spiking neuron in the main text.

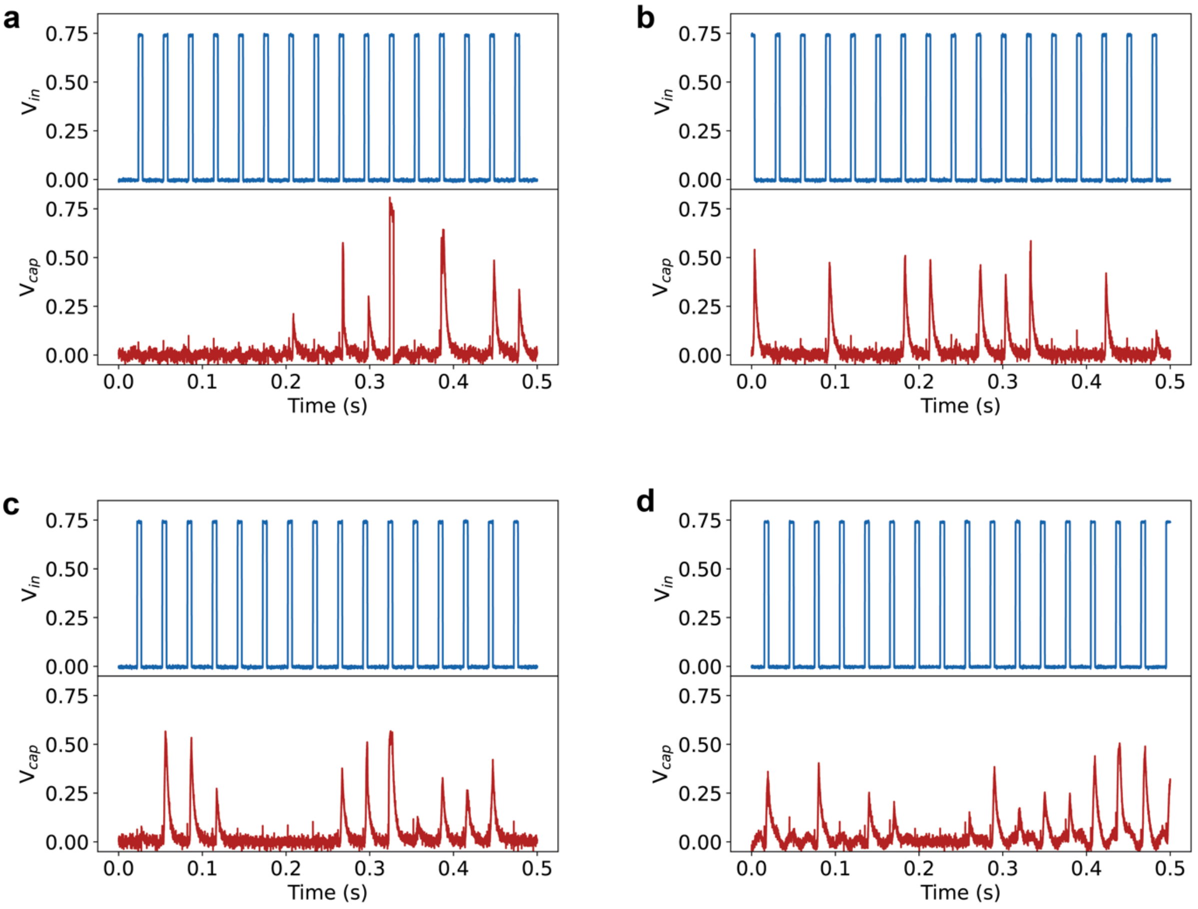

*Figure S7. Four different measurements of the spiking neuron. In the measurements in **(a)**, **(b)**, **(c)**, and **(d)** the same voltage profile, with 5 ms pulses of 750 mV, was applied to the neuron with a 33 Hz frequency. In all cases, this resulted in stochastic spiking by the neuron.*

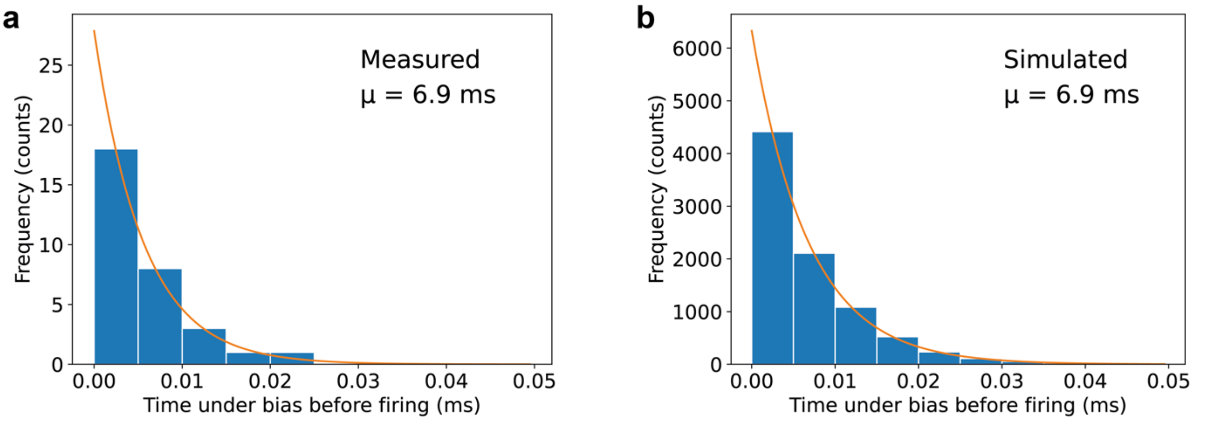

*Figure S8. **(a)** Histogram of the time under bias before firing of the neuron based on the measurements in Figure S7. Spiking by the neuron was defined as the moment when the capacitor voltage exceeds 200 mV. The mean of the distribution is 6.9 ms. **(b)** The same histogram of spiking by the neuron compiled from simulated data to validate the model. We obtain the same mean of 6.9 ms of applied bias before spiking by the neuron.*

Supplementary Note 1. Modeling of stochastic and deterministic neurons

The simulated data in Figure 3 was obtained with experimentally determined resistances and capacitance. For the stochastic neurons, a switching time was drawn from a Poisson distribution with the experimentally determined mean of 6.9 ms from Figure S8, using a random number generator. Voltage pulses are applied in the simulation, which causes a voltage buildup on the capacitor with an RC constant of $300\ \mathrm{pF} \times 1\ \mathrm{G\Omega} = 0.3\ \mathrm{s}$. The time under bias is then tracked until the switching time is reached. At this point, the RC constant decreases significantly to $300\ \mathrm{pF} \times 3\ \mathrm{M\Omega} = 0.9\ \mathrm{ms}$ due to the resistance change of the resistive switch. After the voltage is removed, the capacitor discharges through a 10 MΩ resistor and a new switching time is drawn from the Poisson distribution. The same procedure is followed for deterministic neurons, but with a switching time always set to 6.9 ms. For validation of the model, we simulated the spiking of a neuron using the same input voltage profile as for the measurements in Figure S7, but over a longer period of 500 seconds, constituting 16,666 applied voltage pulses. A histogram of the time under bias before spiking by the neuron based on the simulation is given in Figure S8b. The mean obtained from the simulation of 6.9 ms agrees with the experimentally obtained mean of 6.9 ms.

To determine the representation error of the neuron populations in Figure 3d, the population code of the neuron populations, *i.e.,* the cumulative sum of spikes for each applied pulse, is compared to the ideal population code. For stochastic neurons, the chance of the neurons outputting a spike is $P(5\ ms) = 1 - e^{-\frac{5\ ms}{6.9\ ms}} \approx 0.52$ for each 5 ms input voltage pulse. In the ideal case, the mean number of spikes per neuron in a population is, therefore, equal to 0.52 multiplied by the number of applied pulses. Figure S9a compares the ideal mean number of spikes with that of a simulation of stochastic neuron populations of different sizes. Because of the non-zero chance of spiking by the stochastic neuron for each applied pulse, the population can capture all applied voltage pulses. For larger population sizes, the mean of the pulses approaches that of the ideal case. The deterministic neurons always spike after 6.9 ms of bias, or every second pulse.

Because of the deterministic nature of the neuron, additional spikes are never output by deterministic populations for uneven numbers of applied pulses, as illustrated by Figure S9b.

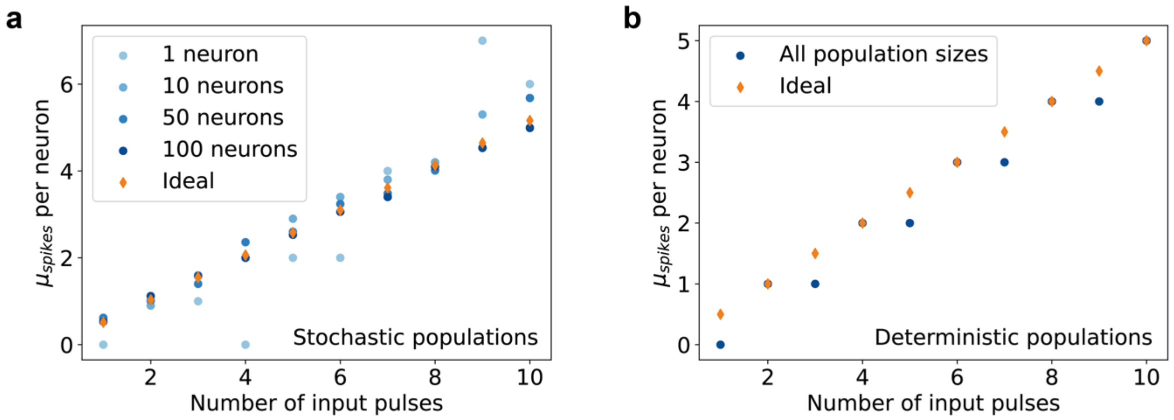

Figure S9. The mean number of spikes per neuron of the stochastic and deterministic neuron populations, compared to their respective ideal mean number of spikes. ***(a)*** *The mean number of spikes per number of stochastic populations of different sizes. Owing to the stochastic nature of the spiking, the average number of spikes converges to the ideal case for all applied pulses as the population size increases.* ***(b)*** *The mean number of spikes of deterministic populations. By definition, the mean does not change for different population sizes. The mean does not increase for uneven number pulses but matches perfectly with the ideal mean for even numbers of applied pulses.*

To obtain the representation error from the simulations, we take the Euclidian norm of the difference between the ideal and the simulated mean numbers of spikes for each population, $E = \sqrt{\sum_{i=1}^{10}(\mu_{i,simulated} - \mu_{i,ideal})^2}$, where *i* refers to the applied pulse number and μ is the mean number of spikes per neuron. To obtain the results shown in Figure 3d, we repeated the simulations 1000 times for neuron populations of 1 and up to 100 neurons. The average representation error is shown in the figure.

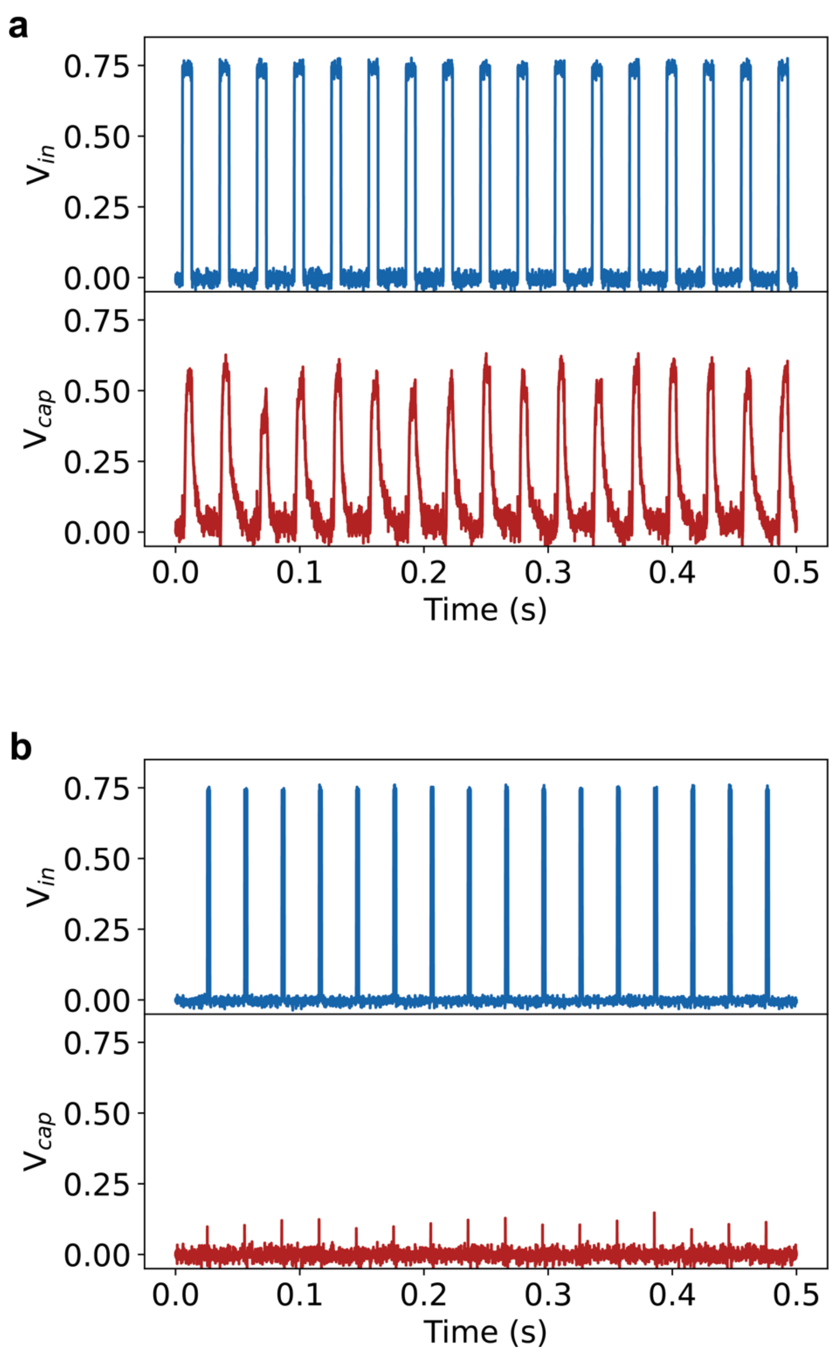

*Figure S10. Stimulation of the neuron with 750 mV pulses with pulse durations of 7.5 ms in **(a)**, which leads to firing with every applied pulse and with a duration of 2 ms in **(b)**, which leads to no firing of the neuron.*

Supplementary Note 2. Scaling of the spiking neuron

There are two main limitations of further scaling of the neuron. First, the RC constant should remain high enough to prevent excessive charge buildup on the capacitor during the stimulation phase. To estimate scaling limitations, we set a limit of 100 mV of voltage buildup during stimulation of the neuron, while the resistive switch is still in the OFF-state. A voltage buildup of 100 mV is still easy to distinguish from a firing event where the voltage rises to several hundreds of millivolts, as in the measurements in Figure S7. Assuming the same input pulses of 750 mV and 5 ms in length as in Figure 2c, the RC constant should then be at least $RC = -\frac{t}{\ln\left(1-\frac{V(t)}{V_{supply}}\right)} = -\frac{5 \times 10^{-3} s}{\ln\left(1-\frac{100\ mV}{750\ mV}\right)} \approx 35\ ms$. Further downscaling of the resistive switch should linearly increase its low-conductance state resistance. Balancing this resistance with the series capacitance to satisfy the constraint on the RC constant allows for easy scaling of the neuron.

Even if the low-conductance state resistance is not reduced further as the resistive switch is scaled, due to a parasitic resistance in the circuit, for example, the capacitance can still be reduced by approximately an order of magnitude, to $C = \frac{.035\ s}{10^9 \Omega} = 35\ pF$, assuming an OFF-state resistance of 1 GΩ extracted from the I-V curve in Figure 1b. Assuming that the reduction in the capacitance means that the capacitor is now always fully charged with a firing event, this would reduce the energy consumption of the neuron to $E = \frac{1}{2} \times C \times V^2 = \frac{1}{2} \times 35 \times 10^{-12} F \times (0.75\ V)^2 \approx 9.8\ pJ$. Even in this upper limit, the energy consumption of the neuron would be close to that of the most energy-efficient silicon neurons.[4]

A second limitation is that the amount of charge on the capacitor should be large enough to aid the resetting of the resistive switch after firing. From Figure S5a we see that the device is in the high resistive state right after the voltage is turned off. Thus, the switching time is <1 ms. The neuron would work in a similar way with a switching time 2-3 orders of magnitude slower, which means that a capacitance 2-3 orders of magnitude smaller would suffice, in the hundreds of femtofarad regime. Common CMOS technology utilizes

architectures that can fabricate capacitors on this scale with very small device footprint.[5]

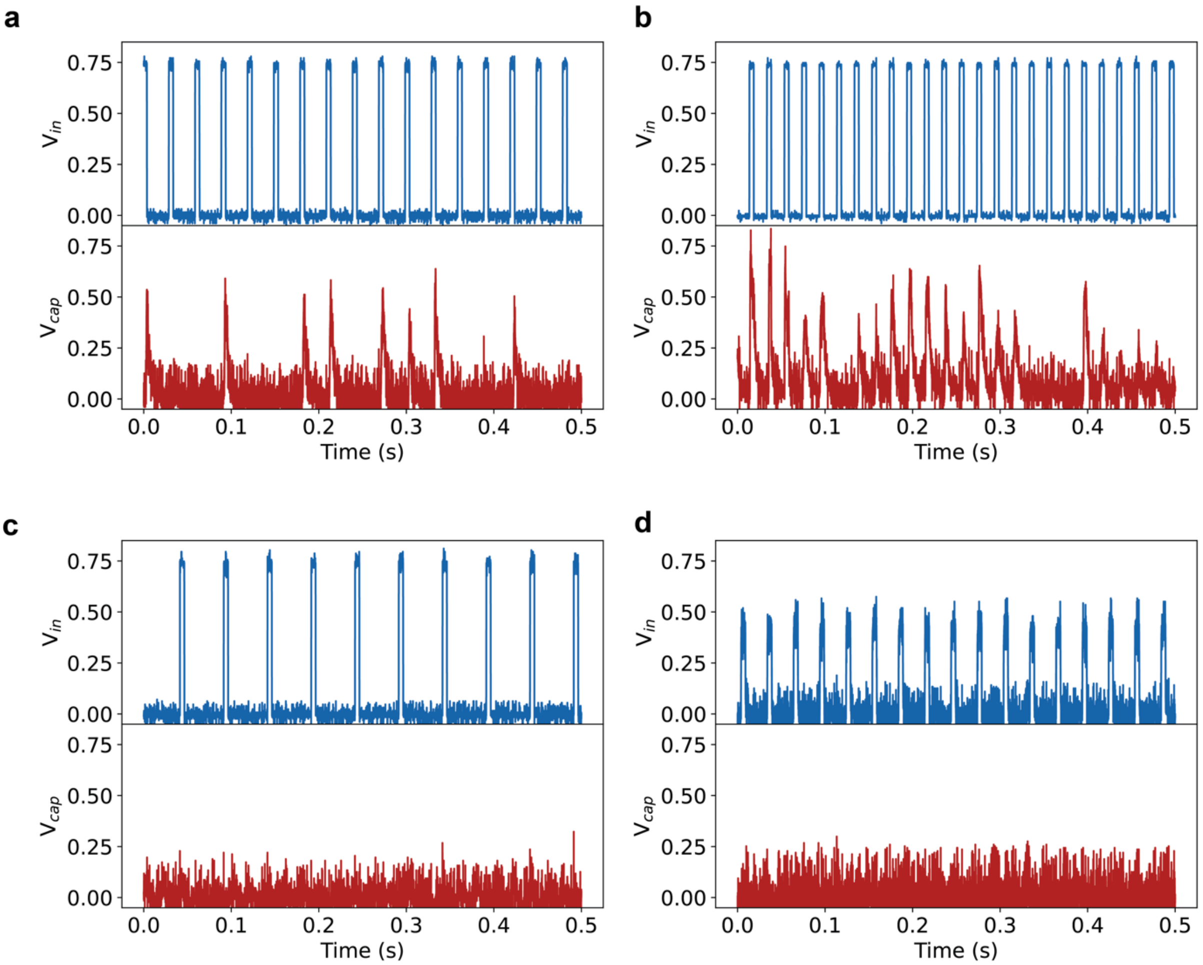

Figure S11. ***(a)*** *Figure 2c,* ***(b)*** *Figure 3a,* ***(c)*** *Figure 3b and* ***(d)*** *Figure 3c before smoothing and removal of the 50 Hz signal of the AC mains. Comparing these figures with the figures in the main text shows that the noise is removed without distorting the measured signal.*

References

1 S. H. Jo, K.-H. Kim and W. Lu, *Nano Lett.*, 2009, **9**, 496–500.

2 X. Zhu, J. Lee and W. D. Lu, *Advanced Materials*, 2017, **29**, 1–8.

3 H. Luo, L. Lu, J. Zhang, Y. Yun, S. Jiang, Y. Tian, Z. Guo, S. Zhao, W. Wei, W. Li, B.

Hu, R. Wang, S. Li, M. Chen and C. Li, *J. Phys. Chem. Lett.*, 2024, **15**, 2453–2461.

4 A. Rubino, M. Payvand and G. Indiveri, in *2019 26th IEEE International Conference on Electronics, Circuits and Systems (ICECS)*, IEEE, Genoa, Italy, 2019, pp. 458–461.

5 M. Brunet and P. Kleimann, *IEEE Trans. Power Electron.*, 2013, **28**, 4440–4448.
